# Hybrid MXene-Graphene/Hexagonal Boron Nitride Structures: Electronic and Molecular Adsorption Properties

**DOI:** 10.3390/nano12162739

**Published:** 2022-08-10

**Authors:** Fawziah Alhajri, Mohamed M. Fadlallah, Amal Alkhaldi, Ahmed A. Maarouf

**Affiliations:** 1 Department of Physics, Science College, Imam Abdulrahman Bin Faisal University, Jubail 3196, Saudi Arabia; 2 Physics Department, Faculty of Science, Benha University, Benha 13518, Egypt; 3 Department of Physics, Faculty of Basic Sciences, German University in Cairo, New Cairo City 11835, Egypt

**Keywords:** 2D materials, DFT, hybrid nanosheets, MXenes, molecular adsorption, graphene, hexagonal boron nitride

## Abstract

Recent advances in experimental techniques allow for the fabrication of hybrid structures. Here, we study the electronic and molecular adsorption properties of the graphene (G)/hexagonal boron nitride (*h*-BN)-MXenes (Mo${}_{2}$C) hybrid nanosheets. We use first-principles calculations to explore the structure and electronic properties of the hybrid structures of G-2H-Mo${}_{2}$C and *h*-BN-2H-Mo${}_{2}$C with two different oxygen terminations of the Mo${}_{2}$C surface. The embedding of G or *h*-BN patches creates structural defects at the patch-Mo${}_{2}$C border and adds new states in the vicinity of the Fermi energy. Since this can be utilized for molecular adsorption and/or sensing, we investigate the ability of the G-M-O1 and BN-M-O1 hybrid structures to adsorb twelve molecules. Generally, the adsorption on the hybrid systems is significantly higher than on the pristine systems, except for N${}_{2}$ and H${}_{2}$, which are weakly adsorbed on all systems. We find that OH, NO, NO${}_{2}$, and SO${}_{2}$ are chemisorbed on the hybrid systems. COOH may be chemisorbed, or it may dissociate depending on its location at the edge between the G/*h*-BN and the MXene. NH${}_{3}$ is chemisorbed/physisorbed on the BN/G-M-O1 systems. CO, H${}_{2}$S, CO${}_{2}$, and CH${}_{4}$ are physisorbed on the hybrid systems. Our results indicate that the studied hybrid systems can be used for molecular filtration/sensing and catalysis.

## 1. Introduction

Research on two-dimensional (2D) materials has been growing rapidly since the successful exfoliation of graphene (G) [1]. The reduced dimensionality changes a material’s behavior compared to its bulk properties, as we see in the transition from the metallic behavior of graphite (3D) to the semimetallic behavior of graphene (2D), or to the semiconducting or metallic behavior of carbon nanotubes (1D). Such low-dimensional systems generate a lot of interest from both fundamental research and application points of view [2]. 2D materials have attracted considerable attention during the past few years because of their unique physical and chemical properties and various potential applications [2,3]. For example, these materials may be used in ultra-thin integrated circuitry required for modern electronics, optoelectronics, sensors, catalysis, and energy storage devices [4]. 2D world materials can be divided into single-atom nanosheets (e.g., silicene [5], and germanene [6]), two-atom nanosheets (e.g., hexagonal boron nitride (*h*-BN) [7], CN [8], BeN${}_{4}$ [9], BeO [10], and transition metal chalcogenides [11,12,13]), and three or more-atom nanosheets (e.g., BCN [14,15], ZnSnN${}_{2}$ [16], MoSi${}_{2}$N${}_{4}$ [17,18,19], and transition metal carbides and nitrides [20]).

MXenes are 2D transition metal carbides and nitrides [20,21,22]. Their chemical compositions are given by the formula M${}_{n+1}$A${}_{n}$ (*n* = 1,2,3), where M is transition metal, and A can be carbon or nitrogen [23]. MXenes have been proposed for energy storage [24], sensors [25], and many other applications [23,26,27]. The surfaces of MXene layers may be chemically terminated/functionalized with certain groups, such as O, F, or OH to avoid dangling bonds (which changes their general formula to M${}_{n+1}$A${}_{n}$X, where X represents groups 16 and 17 elements). The tunable electronic properties, excellent ionic conductivity, high Young’s moduli, and the flexible layer spacing of MXenes make them ideal for many applications [28,29].

A heterostructure comprising two or more dissimilar 2D materials have very different properties from its component materials. Heterostructures are thus emerging as new exciting candidates in materials research [2]. They can exist in layered form, where layers of different 2D materials are held together by van der Waals (vdW) forces. They can also be formed when different 2D materials are combined in the same monolayer, forming a lateral 2D heterostructure [30], or an embedded 2D heterostructure [31]. Heterostructures may exhibit exciting electronic properties and a high potential for use in nanodevices [32,33,34]. The MXene Ti${}_{2}$CX${}_{2}$ (X = F, O, and OH)/graphene (G) heterostructures have been successfully fabricated and can be used as electrodes for Li- and Mg-ion batteries [35,36,37]. Ti${}_{3}$C${}_{2}$T${}_{x}$/*h*-BN/Ti${}_{3}$C${}_{2}$T${}_{x}$ heterostructures have been studied as a supercapacitor material [38]. Porous MXene-Polyimide/nanocellulose hybrids have been used for electromagnetic interference shielding [39,40].

The interaction between G and MXenes has been investigated using first-principles calculations for the cases Ti${}_{3}$C${}_{2}$X${}_{2}$ with different terminal groups, X=O, OH, and F [41]. G with MXene monolayers (M${}_{2}$CX${}_{2}$ where M = Sc, Ti, V and X = OH, O) heterostructures have been studied for Li-battery applications [42]. Furthermore, the G-CN/M${}_{2}$CO${}_{2}$ (M = Hf, Zr, and Sc) heterostructures not only exhibit favorable structure stability but also show potential for photocatalytic degradation of volatile organic compounds [43]. The Ti${}_{3}$C${}_{2}$X${}_{x}$ MXene/G hybrid structure has been synthesized and used for NH${}_{3}$ gas sensing [44] and for highly efficient electrocatalytic hydrogen evolution [45]. The hybrids of Mo${}_{2}$C anchored on G sheets have been studied as anode materials for lithium-ion lithium storage [46].

In this work, we use first-principles calculations to investigate the properties of hybrid structures made from G and h-BN patches inside monolayer 2H-Mo${}_{2}$C. We study their structural and electronic properties with two different oxygen terminations of the Mo${}_{2}$C layer [3,23]. We also explore the adsorption of 12 molecules (H${}_{2}$, N${}_{2}$, CO, OH, NO, CO${}_{2}$, NO${}_{2}$, H${}_{2}$S, SO${}_{2}$, NH${}_{3}$, COOH and CH${}_{4}$) on the hybrid structures.

## 2. Computational Method

First-principle calculations based on density functional theory (DFT) are used within the projector-augmented wave method [47], which is implemented in the Quantum Espresso package [48]. The exchange-correlation interaction is described by the generalized gradient approximation (GGA) via the Perdew-Burke-Ernzerhof (PBE) functional [49]. A 45 Ry energy cut-off is utilized, and a 6 × 6 2H-Mo${}_{2}$C supercell is used as the base MXene system, with a vacuum space of 20 Å to avoid any interaction between periodic images. All systems are fully relaxed (volume and ionic position) until the forces on the atoms become less than 0.001 Ry/Bohr. Van der Waals interactions (vdW-DF) [50,51] are included in our study. Brillouin zone integrations are performed using the tetrahedron method with Blöchl corrections and a 9 × 9 × 1 *k*-point grid to calculate the density of states. Charge transfer from/to the MXene systems are determined by calculating the Löwdin charges. Initial spin-polarized calculations show that all considered structures are non-magnetic.

## 3. Results and Discussions

**Figure 1:**
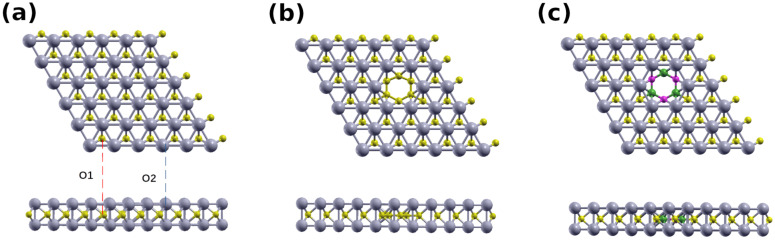
The top and side views of the unterminated (**a**) M, (**b**) G-M, and (**c**) BN-M systems. The projections of the terminations O1 and O2 are shown in (**a**). Mo, C, B, and N atoms are shown in gray, yellow, green, and pink, respectively.

To properly set the scene for our investigation of the hybrid structures, we first calculate the properties of single-layer MXenes (Figure 1). The single-layer MXene structure can be viewed as a 2D triangular lattice of X atoms sandwiched between two triangular lattices of M atoms, similar to single-layer transition metal dichalcogenides [52,53]. We investigate multiple Mo${}_{2}$C structures: pristine (M), G-Mo${}_{2}$C hybrid (G-M), and *h*-BN-Mo${}_{2}$C hybrid (BN-M), each with two different oxygen terminations (Figure 1a). The systems with the first termination (O1) have an oxygen atom atop the Mo layer between 3 Mo atoms (above a carbon site), while systems with the second termination (O2) have the oxygen atom atop a Mo atom. According to prior work, functional groups seem to prefer hollow sites over metal-atom sites [26]. To assess the stability of the G-M-O1, G-M-O2, BN-M-O1, and BN-M-O2, we calculate their formation energies ($E_{form}$) using the formula:

(1) $$E_{form}=\frac{\left(E_{h}-E_{X}-E_{Y}\right)}{n_{h}},$$

where $E_{h}$ and $E_{X}$ ($E_{X}=n_{\mathrm{Mo}}E_{\mathrm{Mo}}+n_{C}E_{C}+n_{O}E_{O}$) are the total energies of the hybrid sheet and its isolated constituent atoms, respectively, with a total number of atoms $n_{h}$. $E_{Y}=n_{C}E_{C}$ for the G-patch and $E_{Y}=n_{B}E_{B}+n_{N}E_{N}$ for the *h*-BN-patch. $E_{z}$ and $n_{z}$ (*z* = Mo, C, O, B, and N) are the energy of the isolated atom and the number of the *z* atoms in the hybrid or patch. The calculated $E_{form}$ values are summarized in Table 1. As we see, the values of $E_{form}$ indicate that these structures are stable. Furthermore, we notice that the O1 structures are more stable than their O2 counterparts, as reported in previous work [54].

**Figure 2:**
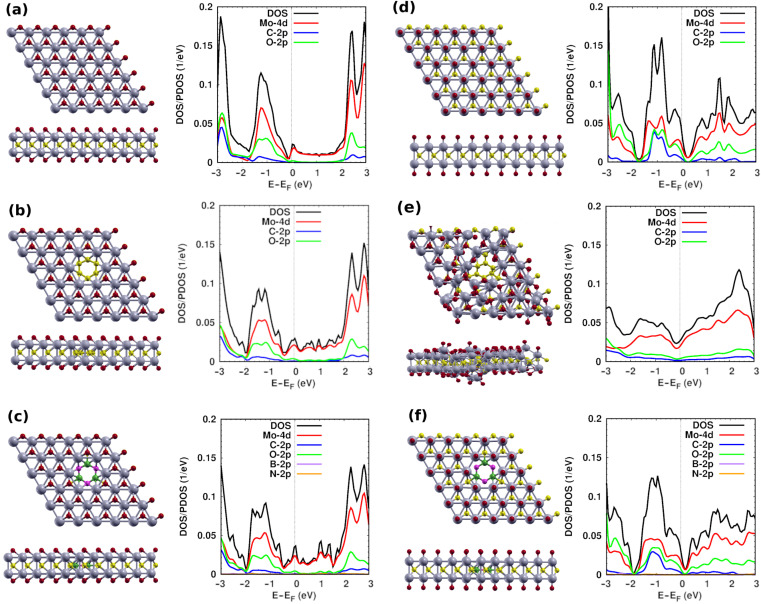
The optimized structures with O termination, top and side views, and DOS/PDOS of (**a**) M-O1, (**b**) G-M-O1, (**c**) BN-M-O1, (**d**) M-O2, (**e**) G-M-O2, and (**f**) BN-M-O2. Mo, C, O, B, and N atoms are shown in gray, yellow, red, green, and pink, respectively.

In the M-O1 system, we find the lattice constant of the supercell, and the bond lengths Mo-Mo, Mo-C, and Mo-O are 6.07 Å, 2.84 Å, 2.17 Å and 2.05 Å, respectively, (see Table 1). The calculated values of the lattice constant and bond lengths agree well with those in previous reports (lattice constant = 5.64–6.00 Å, Mo-Mo = 2.73 Å, Mo-C = 2.14 Å, Mo-0 = 2.06 Å) [3,23,55,56]. The electronic properties of the pristine M-O1 show that this mono-layered 2D material is metallic. The total and projected densities of states (DOS and PDOS, respectively) are shown in Figure 2a. At the Fermi energy and above it, the Mo-4d states dominate the spectrum. The O-2p states contribute from −0.25 eV below the Fermi energy to 2 eV above it. The C-2p states contribute far from the Fermi energy in the range (−2.0,−3.0) eV and above 2.0 eV. The DOS/PDOS spectra agree with recent literature [55]. The MXenes provide more 4d electrons because of the two metal atoms in the *primitive* cell. Therefore, pristine Mo${}_{2}$C are metallic structures with states near the Fermi energy, making them superior candidates for molecular adsorption.

We now discuss our hybrid systems (G and *h*-BN inside the M-O1, Figure 2b,c. The embedded graphene comprises a patch made up of 6 carbon atoms inside M-O1 after removing 2 Mo, 6 O atoms, and 6 C atoms, then adding a carbon hexagon. Structural relaxation gives us a lattice constant of 6.07 Å similar to that of M-O1. The hybrid G-M-O1 structure has a minor symmetric distortion at the Mo sites surrounding the patch where the bond lengths slightly change (see Table 1), and the distance between Mo-patch ($d_{4}$ and C-patch ($d_{5}$) is 2.26 Å and 2.35 Å, respectively. The atoms moved away from their original positions to release the stress in the system, leading to the slight increase or decrease of the bond length as shown in Figure 2b. The DOS of G-M-O1 slightly shifts towards low energy as compared to the pristine structure. The contribution of C states is still tiny as compared to other states, such as in the energy range from −1.9 eV to 3.0 eV. The DOS in the energy range from 0.2 eV to 2.7 eV has many peaks because of the effect of the carbon ring and Mo atoms around the patch. The DOS at the Fermi energy is higher than the corresponding value for the pristine structure, which indicates that the G patch increases the conductivity of M-O1.

The BN-M-O1 system is constructed by removing 2 Mo, 6 O, and 3 C atoms, then adding a BN hexagon (Figure 2c). After structural relaxation, the lattice constant does not change. The bond lengths slightly change as compared to the previous structures to release the stress in the system (Table 1), and $d_{4}$ and $d_{5}$ are 2.26 Å and 1.73 Å, respectively. The DOS shifts towards the low energy, and the DOS in the energy range from 0.22 eV to 2.7 eV has many peaks due to the contribution of the B and N state effect of Mo atoms around the patch.

Turning to the pristine M-O2 system, Figure 2d, the lattice constant of a 6 × 6 supercell is similar to that of M-O1. The system has 72 O termination atoms. The Mo-Mo and Mo-C bond lengths increase to 2.93 Å, and 2.23 Å, respectively, while the Mo-O bond length decreases to 1.72 Å. All calculated values of the lattice constants and bond lengths agree well with those in previous reports (Mo-C = 2–2.5 Å, Mo-O = 1.9–2.3 Å) [3,23,56]. The DOS shows the metallic behavior of the M-O2 sheet. Mo states dominate from −2.8 eV to 3.0 eV, with some contributions from the O states. The C states have large contributions from −1.8 eV to −0.8 eV. The contribution below −2.0 eV is small as compared to the corresponding states in pristine M-O1. The DOS in the energy range from 0.2 eV to 2.7 eV is not constant as compared to the DOS of M-O1.

The relaxed G-M-O2 structure, Figure 2e, exhibits more distortion than the G-M-O1 structure, especially at the interface between the G patch and the M-O2. The lattice constant of hybrid-O2 structures does not change as compared to the lattice constant of M-O2 system. The bond lengths Mo-Mo, and Mo-C decrease to 2.86 Å, 2.18 Å, but that of Mo-O increases to 1.95 Å as compared to M-O2 system. The $d_{4}$ and $d_{5}$ are 2.20 Å and 1.44 Å, respectively, less than the corresponding values in G-M-O1 structures. The graphene patch increases the density of states near the Fermi energy. The contributions of O states and C states are small as compared to Mo states and the corresponding states in the pristine structure. The BN-M-O2 structure does not suffer from any significant deformation after structural optimization (Figure 2f). The bond length of Mo–Mo (around the *h*-BN patch) decreases to 2.86 Å, and Mo-C increases to 2.24 Å, but Mo-O does not change as compared to M-O2 structure. The $d_{4}$ and $d_{5}$ are 2.40 Å and 1.65 Å, respectively, (Table 1). The DOS of BN-M-O2 is very similar to that of the pristine structure. It has been reported that the M-O2 structure is less stable than M-O1 [54]. Furthermore, the large distortions observed in the G-M-O2 structure may render the molecular adsorption on it surface highly dependent on the adsorption site. Therefore, we limit our molecular adsorption studies to the G/BN-M-O1 structures [54].

### Gas Adsorption on MXenes

**Figure 3:**
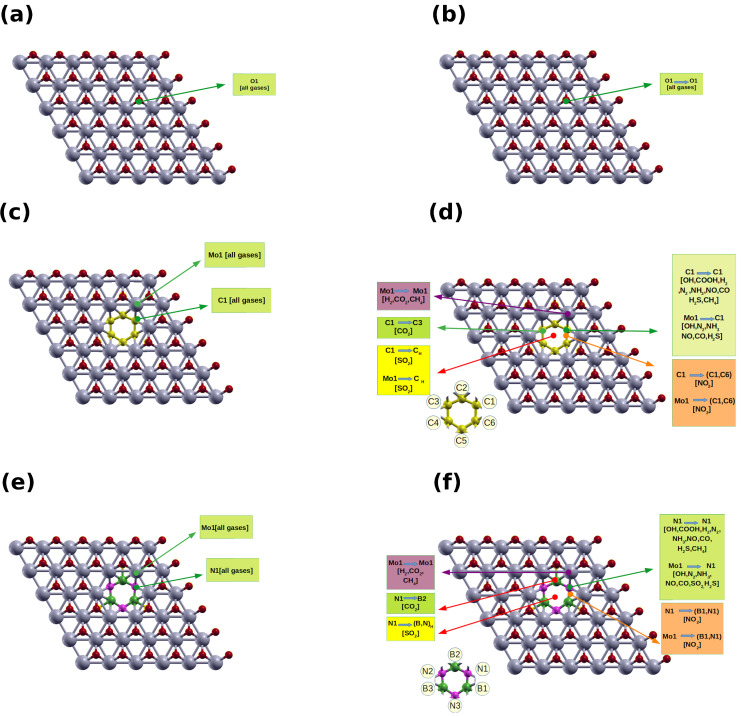
Adsorption locations of various molecules on (**a**,**b**) pristine M-O1, (**c**,**d**) G-M-O1, and (**e**,**f**) BN-M-O1. C${}_{H}$ and (B,N)${}_{H}$ refer to the center of the patch.Mo, C, O, B, and N atoms are shown in gray, yellow, red, green, and pink, respectively.

We started the structural relaxation of the adsorbent-sheet systems on three distinct adsorption sites. On the pristine structure, we placed the adsorbent close to an oxygen atom (position O1). In the hybrid structures, we have two distinct starting points: above a C/N atom of the (G/*h*-BN hexagon (points C1/N1), and above the Mo atom at the edge of the patch (point Mo1, Figure 3c,e).

We consider 12 small molecules, and divide them into four groups, diatomic molecules (OH, NO, CO, N${}_{2}$, H${}_{2}$), triatomic molecules (NO${}_{2}$, H${}_{2}$S, SO${}_{2}$, CO${}_{2}$), tetratomic molecules (COOH, NH${}_{3}$), and polyatomic molecules (CH${}_{4}$). The adsorption energy is calculated using the formula:

(2) $$E_{ad}=E_{\mathrm{sheet}+\mathrm{adsorbate}}-E_{\mathrm{sheet}}-E_{\mathrm{adsorbate}},$$

where $E_{\mathrm{sheet}+\mathrm{adsorbate}}$ is the total energy of the sheet with the adsorbate, $E_{\mathrm{sheet}}$ is the total energy of the pristine or hybrid sheet, and $E_{\mathrm{adsorbate}}$ is the total energy of the isolated molecule.

**Figure 4:**
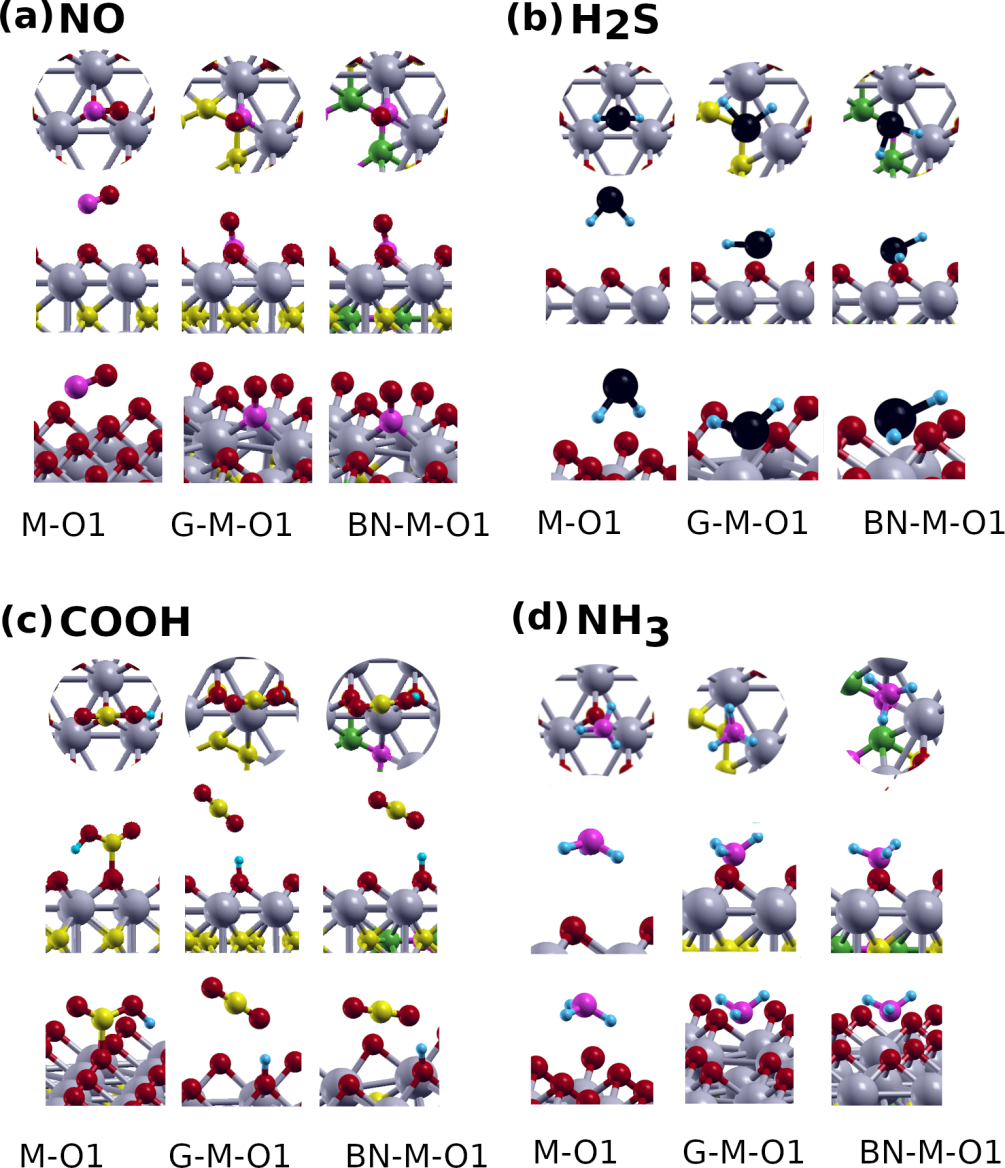
The optimized structures of (**a**) NO, (**b**) H${}_{2}$S, (**c**) COOH, and (**d**) NH${}_{3}$ adsorbed molecules on M-O1, G-M-O1, and BN-M-O1 nanosheets in different side views. Mo, C, O, B, and N, S, and H atoms are shown in gray, yellow, red, green, pink, black, and blue, respectively.

In order to find the minimum configuration for the adsorbent, the diatomic molecules are initially placed at (O1) (Figure 3a). After geometrical optimization, the molecules do not change their positions. By analyzing the structural data for diatomic molecules, We found that the OH bond length of the isolated OH molecule is 0.98 Å, which is close to the literature value of (0.97 Å [57]). It slightly increases to 0.99 Å after adsorption on all the pristine and hybrid sheets. The bond lengths of the isolated NO, CO are 1.16 Å, and 1.14 Å, respectively, which match previous reports (1.15 Å,and 1.13 Å, respectively, [58,59]). The NO bond slightly decreases to 1.12 Å (Figure 4a) on M-O1 and increases to 1.20 Å on G/BN-M-O1 (Figure 4a). The CO bond is the same as for the isolated CO (1.13 Å), and increases to 1.17 Å on G/BN-M-O1. The bond lengths of the isolated N${}_{2}$ and H${}_{2}$ are 1.10 Å and 0.75 Å, respectively, which are close to those in previous reports (1.09 Å and 0.74 Å, respectively, [58]). These become 1.11 Å and 0.75 Å, respectively, on M-O1, and 1.11 Å and 0.76 Å, respectively, on G/BN-M-O1.

The adsorption energy $E_{ad}$ of the most stable configurations was calculated by Equation (2). The detailed results of all the studied cases are presented in Table 2. Energies up to ∼2 eV indicate physisorption, while higher values indicate chemisorption [60] Calculations of $E_{ad}$ and ΔQ (e) of diatomic molecules on M-O1 show that OH, NO, and CO have the largest energies, which can be explained by the polarity of oxide molecules. Thus, OH and NO are chemisorbed on M-O1 while CO, N${}_{2}$, and H${}_{2}$ are physisorbed. The OH molecule stands at a distance of 1.45 Å above the M-O1 surface, with its O atom facing the sheet. For NO and CO, N and C are the atoms of contact with the sheet, respectively. As for the bond distance of NO (O-N${}_{m}$), it is 2.05 Å, while for CO the distance (O-C${}_{m}$) is 4.17 Å.

For adsorption on G-M-O1 hybrid, all the diatomic molecules starting from the C1 position end up in the same place after structural optimization (C1 → C1). When we start the relaxation from the Mo1 position, the diatomic molecules move to C1 (Mo1 → C1) except the H${}_{2}$ molecule, which stays in Mo1 (Mo1 → Mo1), (Figure 3c,d).

For the BN-M-O1 system, all adsorbed diatomic molecules starting from the N1 position do not change their positions after structural relaxation (N1 → N1). These molecules at the Mo1 position move to the N1 position after optimization (Mo1 → N1), except for H${}_{2}$, which stays at Mo1 (Mo1 → Mo1) (Figure 3e,f). The available theoretical and experimental studies show that the adsorption of diatomic molecules on pristine (G) [61,63,64] and pristine *h*-BN)[61,65,66,67] are smaller than the corresponding value on the hybrid sheets, except for H${}_{2}$ molecule, (see Table 2). The E${}_{ad}$ in the case of the G or *h*-BN hybrid is much higher than in that of M-O1, pristine G, and pristine *h*-BN for all molecules. This is because of electrostatic irregularities at the border between the patch and the original sheet. The molecules are adsorbed at the same atom as in the case of M-O1, and the distance between the molecules and the sheet becomes larger as compared to the M-O1 sheet except for CO (see Table 2).

Regarding triatomic molecules, the initial and final positions of the adsorbed molecules on the different sheets are shown in (Figure 3) and listed in Table 3. For the M-O1 system, and for all triatomic molecules, we find that the final position is the same as the initial position (Figure 3a,b). For the hybrid systems, an NO${}_{2}$ molecule starting at the C1 or Mo1 positions ends up at the midpoint between C1 and C6 (C1 → C1-C6). The N–O bond length and the O–N–O angle of the isolated molecule are 1.21 Å and 133.5${}^{\circ }$, respectively, which agree with values reported in the literature (1.20 Å and 134.3${}^{\circ }$[68]). After adsorption, these change to 1.17 Å and 145.62${}^{\circ }$ for the pristine sheet, and 1.26 Å and 118.25${}^{\circ }$ for G-M-O1 and BN-M-O1 sheets. For SO${}_{2}$, the S–O bond length and the O–S–O angle are 1.45 Å and 119.5${}^{\circ }$, respectively, which also agree with the corresponding values in the literature (1.43 Å and 119${}^{\circ }$[69]). These become 1.45 Å and 118.57${}^{\circ }$ on the pristine sheet, and 1.62 Å and 108.97${}^{\circ }$ on the hybrid systems G-M-O1 and BN-M-O1.

The changes occurring in the H${}_{2}$S and CO${}_{2}$ molecules are noticeably less. The calculated H–S bond length and the H–S–H angle are 1.35 Å and 61.61${}^{\circ }$ on the M-O1 sheet (Figure 4b), and 1.37 Å and 94.52${}^{\circ }$ for the hybrid sheets (Figure 4b), compared to 1.35 Å and 91.7${}^{\circ }$ for the isolated molecule (literature values are 1.34 Å and 92.1${}^{\circ }$[70]). For CO${}_{2}$, we find that C–O bond length and the O–C–O angle to be 1.17 Å and 179.86${}^{\circ }$ for the pristine sheet, and 1.45 Å and 178.47${}^{\circ }$ for the hybrid sheets, compared to 1.17 Å and 180${}^{\circ }$ for the free molecule (literature values are 1.16 Å and 180${}^{\circ }$ [68]).

We notice that in the 4 cases, the bond length increases upon adsorption on the hybrid systems due to the redistribution of the electronic charge. For NO${}_{2}$ and SO${}_{2}$, we see large adsorption energies and small charge transfers, indicating that the bonds formed are more covalent in nature, which can be described as chemisorption. For H${}_{2}$S and CO${}_{2}$, the energies are small, but we see a moderate charge transfer, with longer bonds with the hybrid Mo atom, indicating physisorption. We also notice that the adsorption occurs through the N and O atoms for the case of NO${}_{2}$ and SO${}_{2}$, respectively, while for H${}_{2}$S (Figure 4b) and CO${}_{2}$, it occurs through the H and C atoms, respectively. This can be explained by the difference in electronegativity between the adsorbent atom and the O atom of the sheet. Table 3 shows that NO${}_{2}$ has the highest adsorption energy for all sheets. Compared to the G, *h*-BN, and M-O1 sheets, the hybrid sheets are superior in adsorbing the studied triatomic molecules, with BN-M-O1 offering the highest adsorption. The adsorption energies and the distances between various adsorbents and the underlying systems are shown in Table 3.

Now we consider the adsorption of the tetratomic molecules COOH, and NH${}_{3}$, and the polyatomic molecule CH${}_{4}$ on the pristine and hybrid systems. The COOH molecule is physisorbed on the M-O1 sheet at the C1 location, while it is chemisorbed with a larger energy on the hybrid systems at the same location. If the molecule is positioned at the Mo1 location on either hybrid sheet, it dissociates into a CO${}_{2}$ molecule and a H atom that gets attached to a sheet O atom (Figure 4c). The bond lengths and the angles of the isolated NH${}_{3}$ and CH${}_{4}$ are 1.02Å and 106.67${}^{\circ }$, and 1.09 Å and 110.41${}^{\circ }$, respectively, which match those of previous reports (1.00 Å and 109.47${}^{\circ }$, and 1.09 Å and 109.5${}^{\circ }$, respectively, [76,77]). The bond lengths and the angles of both molecules do not change significantly after adsorption. The optimized location for NH${}_{3}$ is at C1/N1 on G-M-O1/BN-M-O1. For CH${}_{4}$, the molecule stays at its initial locations: C1/Mo1 on G-M-O1, and Mo1 on BN-M-O1.

As expected, the adsorption energy of COOH is larger than those of NH${}_{3}$ and CH${}_{4}$ due to the structure of the molecule which allows for an easier redistribution of charge (Figure 4c). This translates to a closer distance d(Y${}_{s}$-Z${}_{m}$) between the molecule and the hybrid systems (Table 4). As before, and for the three molecules, the adsorption on the hybrid systems is higher than on the pristine system.

## 4. Conclusions

In this paper, we use density functional theory to investigate the structure and electronic properties of the hybrid structures of graphene (G)/2H-Mo${}_{2}$C (G-M), and hexagonal boron nitride ( *h*-BN)/2H-Mo${}_{2}$C (BN-M), with two different O-functionalizations (O1 and O2). The G-M-O1/O2 and BN-M-O1/O2 hybrid sheets are metallic as 2H-MXenes (M-O1 and M-O2). The G and *h*-BN patches create localized states near the Fermi energy, which can be utilized for the adsorption/sensing of some molecules. We thus investigate the ability of G-M-O1 and BN-M-O1 hybrid structures to adsorb twelve molecules: diatomic (OH, NO, CO, N${}_{2}$, H${}_{2}$), triatomic (NO${}_{2}$, SO${}_{2}$, H${}_{2}$S, CO${}_{2}$), and polyatomic (COOH, NH${}_{3}$, and CH${}_{4}$). Generally, the change in the bond lengths and angles of the molecules upon adsorption are larger on the hybrid systems compared to the pristine system (except for H${}_{2}$, which is weakly adsorbed on all systems). This is primarily due to the structural deformations occurring at the borders between the G/ *h*-BN and the MXene regions, which act as electrostatic/covalent traps. We found that OH, NO, NO${}_{2}$, and SO${}_{2}$ are chemisorbed on G-M-O1 and BN-M-O1. COOH may be chemisorbed, or it may dissociate depending on its location at the edge between the G/ *h*-BN and the MXene. NH${}_{3}$ is chemisorbed/physisorbed on the BN/G-M-O1 systems. The other studied molecules are all physisorbed on the hybrid systems. The results of our work indicate that the hybrid nanosheets can be used in catalysis and molecular sensing.

## Figures and Tables

**Table 1:** The calculated formation energy ($E_{form}$ eV), lattice constant (Å), average bond length (Å) of Mo-Mo ($d_{1}$), Mo-C ($d_{2}$), Mo-O ($d_{3}$), Mo-patch ($d_{4}$) and C-patch ($d_{5}$), and the number of O-termination atoms ($N_{O}$).

Sys.	$E_{\mathrm{form}}$	Lattice Constant	$d_{1}$	$d_{2}$	$d_{3}$	$d_{4}$	$d_{5}$	$N_{O}$
M-O1	−9.16	6.07	2.84	2.17	2.05	-	-	72
G-M-O1	−9.11	6.07	2.83	2.17	2.08	2.26	2.35	66
BN-M-O1	−9.09	6.07	2.85	2.20	2.04	2.26	1.73	70
M-O2	−8.30	6.07	2.93	2.23	1.72	-	-	72
G-M-O2	−8.82	6.07	2.86	2.18	1.95	2.20	1.44	66
BN-M-O2	−8.30	6.07	2.86	2.24	1.72	2.40	1.65	70

**Table 2:** (X${}_{i}$-X${}_{f}$) refers to the adsorbed site (before-after) relaxation, E${}_{ad}$ is the adsorption energy (eV), the closest distance between the sheet and molecule is d(Y${}_{s}$-Z${}_{m}$) (Å), where Y${}_{s}$ and Z${}_{m}$ indicate the sheet atom and adsorbed-molecule atom, respectively. ΔQ (e) is the charge transfer, which is negative when a charge is transferred from the structure to a molecule. The references in the adsorption energy columns refer to the corresponding values of the gas adsorption on G, and *h*-BN monolayers.

Gas	M-O1				G-M-O1				BN-M-O1			
	**X** ${}_{i}$ **-X** ${}_{f}$	**E** ${}_{\mathrm{ad}}$	Δ **Q**	**d(Y**${}_{s}$-Z${}_{m}$**)**	**X** ${}_{i}$ **-X** ${}_{f}$	**E** ${}_{\mathrm{ad}}$	Δ **Q**	**d(Y** ${}_{s}$ **-Z** ${}_{m}$ **)**	**X** ${}_{i}$ **-X** ${}_{f}$	**E** ${}_{\mathrm{ad}}$	Δ **Q**	**d(Y**${}_{s}$-Z${}_{m}$**)**
OH	O1-O1	−1.15	−0.21	1.45 (O-O${}_{m}$)	C1-C1	−5.48/−0.5 [61]	0.07	2.11 (Mo-O${}_{m}$)	N1-N1	−5.45	0.07/−2.3 [61]	2.11 (Mo-O${}_{m}$)
	–	–	–	–	Mo1-C1	−5.48	0.06	2.11 (Mo-O${}_{m}$)	Mo1-N1	−5.45	0.07	2.11 (Mo-O${}_{m}$)
NO	O1-O1	−1.10	−0.56	2.05 (O-N${}_{m}$)	C1-C1	−2.58	−0.06	2.07 (Mo-N${}_{m}$)	N1-N1	−3.15/−0.026 [62]	−0.06	2.09 (Mo-N${}_{m}$)
	–	–	–	–	Mo1-C1	−2.58	−0.07	2.07 (Mo-N${}_{m}$)	Mo1-N1	−3.15	−0.07	2.09 (Mo-N${}_{m}$)
CO	O1-O1	−0.10	−0.07	4.17 (O-C${}_{m}$)	C1-C1	−1.26/−1.13 [63]	−0.21	2.21 (Mo-C${}_{m}$)	N1-N1	−1.81/−0.02 [62]	−0.21	2.21 (Mo-C${}_{m}$)
	–	–	–	–	Mo1-C1	−1.27	−0.19	2.21 (Mo-C${}_{m}$)	Mo1-N1	−1.81	−0.19	2.22 (Mo-C${}_{m}$)
N${}_{2}$	O1-O1	−0.09	−0.07	3.08 (O-N${}_{m}$)	C1-C1	−0.23	−0.20	3.12 (Mo-N${}_{m}$)	N1-N1	−0.64	−0.21	3.28 (O-N${}_{m}$)
	–	–	–	–	Mo1-C1	−0.24	−0.17	3.03 (Mo-N${}_{m}$)	Mo1-N1	−0.64	−0.18	3.26 (Mo-N${}_{m}$)
H${}_{2}$	O1-O1	−0.05	−0.014	2.46 (O-H${}_{m}$)	C1-C1	−0.07/−0.08 [64]	−0.02	3.58 (O-H${}_{m}$)	N1-N1	−0.09/−0.21 [65]	−0.02	2.90 (Mo-H${}_{m}$)
	–	–	–	–	Mo1-Mo1	−0.08	−0.06	2.69 (O-H${}_{m}$)	Mo1-Mo1	−0.06	−0.02	2.74 (O-H${}_{m}$)

**Table 3:** X${}_{i}$-X${}_{f}$ refers to the adsorbed site before-after relaxation, E${}_{ad}$ is the adsorption energy (eV). The closest distance between the sheet and an adsorbent is d(Y${}_{s}$-Z${}_{m}$) (Å), where Y${}_{s}$ and Z${}_{m}$ indicate the sheet atom and adsorbent atom, respectively, and ΔQ (e) refers the charge transfer where the negative sign means charge is transferred from the structure to an adsorbent. C${}_{H}$ and (B,N)${}_{H}$ refer to the final position located at the hexagonal ring of G and *h*-BN, respectively. The references in the adsorption energy columns refer to the corresponding values of the adsorbent gases on the pristine G and *h*-BN monolayers.

Gas	M-O1				G-M-O1				BN-M-O1			
	**X** ${}_{i}$ **-X** ${}_{f}$	**E** ${}_{\mathrm{ad}}$	Δ **Q**	**d (Y**${}_{s}$-Z${}_{m}$**)**	**X** ${}_{i}$ **-X** ${}_{f}$	**E** ${}_{\mathrm{ad}}$	Δ **Q**	**d (Y** ${}_{s}$ **-Z** ${}_{m}$ **)**	**X** ${}_{i}$ **-X** ${}_{f}$	**E** ${}_{\mathrm{ad}}$	Δ **Q**	**d (Y** ${}_{s}$ **-Z** ${}_{m}$ **)**
NO${}_{2}$	O1-O1	−0.58	−0.59	1.97 (O-N${}_{m}$)	C1-(C1,C6)	−2.46/−2.17 [71]	−0.03	2.21 (Mo-N${}_{m}$)	N1-(B1,N1)	−2.91/−0.03 [62]	−0.03	2.21 (Mo-N${}_{m}$)
	–	–	–	–	Mo1-(C1,C6)	−2.46	−0.04	2.21 (Mo-N${}_{m}$)	Mo1-(B1,N1)	−2.91	−0.04	2.20 (Mo-N${}_{m}$)
SO${}_{2}$	O1-O1	−0.49	−0.06	3.66 (O-O${}_{m}$)	C1-C${}_{H}$	−2.31/−1.97 [72]	−0.12	2.13 (Mo-O${}_{m}$)	N1-(B,N)${}_{H}$	−2.78/−0.08 [67]	−0.13	2.14 (Mo-O${}_{m}$)
	–	–	–	–	Mo1-C${}_{H}$	−2.28	−0.13	2.15 (Mo-O${}_{m}$)	Mo1-N1	−1.93	−0.24	2.15 (Mo-O${}_{m}$)
H${}_{2}$S	O1-O1	−0.26	−0.27	3.46 (O-H${}_{m}$)	C1-C1	−1.19/−0.45 [73]	−0.61	2.78 (Mo-S${}_{m}$)	N1-N1	−1.65/−0.11 [74]	−0.59	2.69 (Mo-S${}_{m}$)
	–	–	–	–	Mo1-C1	−1.22	−0.62	2.72 (Mo-S${}_{m}$)	Mo1-N1	−1.65	−0.61	2.71 (Mo-S${}_{m}$)
CO${}_{2}$	O1-O1	−0.19	−0.06	2.80 (O-C${}_{m}$)	C1-C3	−0.56/-0.32 [75]	−0.32	2.54 (Mo-O${}_{m}$)	N1-B2	−0.78/−0.02 [62]	−0.05	2.95 (Mo-O${}_{m}$)
	–	–	–	–	Mo1-Mo1	−0.12	−0.22	3.07 (O-O${}_{m}$)	Mo1-Mo1	−0.55	−0.05	3.05 (O-O${}_{m}$)

**Table 4:** (X${}_{i}$-X${}_{f}$) refers to the adsorbed site (before-after) relaxation, E${}_{ad}$ is the adsorption energy (eV). The closest distance between the sheet and molecule is d(Y${}_{s}$-Z${}_{m}$) (Å) where Y${}_{s}$ and Z${}_{m}$ indicate the sheet atom and the adsorbed molecule atom, respectively, and ΔQ (e) refers the charge transfer where a negative sign means the charge is transferred from the structure to a molecule. C${}_{H}$ and (B,N)${}_{H}$ mean the final position is at the hexagonal ring of G and *h*-BN, respectively. The “D” indicates the dissociation of the molecule. The references in the adsorption energy columns refer to the corresponding values of the adsorbents on the pristine G and *h*-BN monolayers.

Gas	M-O1				G-M-O1				BN-M-O1			
	**X** ${}_{i}$ **-X** ${}_{f}$	**E** ${}_{\mathrm{ad}}$	Δ **Q**	**d (Y**${}_{s}$-Z${}_{m}$**)**	**X** ${}_{i}$ **-X** ${}_{f}$	**E** ${}_{\mathrm{ad}}$	Δ **Q**	**d (Y**${}_{s}$-Z${}_{m}$**)**	**X** ${}_{i}$ **-X** ${}_{f}$	**E** ${}_{\mathrm{ad}}$	Δ **Q**	**d (Y** ${}_{s}$ **-Z** ${}_{m}$ **)**
COOH	O1-O1	−2.95	−0.45	1.54 (O-C${}_{m}$)	C1-C1	−3.32	−0.24	2.26 (Mo-O${}_{m}$)	N1-N1	−3.11	−0.53	2.41 (Mo-C${}_{m}$)
	–	–	–	–	Mo1-d	D	D	D	Mo1-d	D	D	D
NH${}_{3}$	O1-O1	−0.40	−0.30	2.66 (O-N${}_{m}$)	C1-C1	−1.78/−1.09 [71]	−0.45	2.95 (O-N${}_{m}$)	N1-N1	−2.18/−0.17 [78]	−0.45	2.80 (O-N${}_{m}$)
	–	–	–	–	Mo1-C1	−1.78	−0.46	2.76 (Mo-N${}_{m}$)	Mo1-N1	−2.66	−0.46	2.75 (Mo-N${}_{m}$)
CH${}_{4}$	O1-O1	−0.21	−0.03	2.81 (O-H${}_{m}$)	C1-C1	−0.41/−0.33 [75]	−0.13	2.36 (O-H${}_{m}$)	N1-N1	−0.80/−0.12 [79]	−0.03	2.40 (O-H${}_{m}$)
	–	–	–	–	Mo1-Mo1	−0.24	−0.13	2.60 (O-H${}_{m}$)	Mo1-Mo1	−0.66	−0.04	2.60 (O-H${}_{m}$)

## Data Availability

The data presented in this study are available in article.
